# Identification of the E2F1-RAD51AP1 axis as a key factor in MGMT-methylated GBM TMZ resistance

**DOI:** 10.20892/j.issn.2095-3941.2023.0011

**Published:** 2023-06-05

**Authors:** Junhu Zhou, Fei Tong, Jixing Zhao, Xiaoteng Cui, Yunfei Wang, Guangxiu Wang, Chunsheng Kang, Xiaomin Liu, Qixue Wang

**Affiliations:** 1 Tianjin Medical University General Hospital, Tianjin 300052, China; 2 Tianjin Neurological Institute, Key Laboratory of Neurotrauma, Variation, and Regeneration, Ministry of Education and Tianjin Municipal Government, Tianjin 300052, China; 3 Department of Oncology, Gamma Knife Center, Department of Neurological Surgery, Tianjin Huanhu Hospital, Nankai University, Tianjin 300350, China

**Keywords:** TMZ, EGFRvIII, E2F1, RAD51AP1, MGMT

## Abstract

**Objective:**

Epidermal growth factor receptor variant III (EGFRvIII) is a constitutively-activated mutation of EGFR that contributes to the malignant progression of glioblastoma multiforme (GBM). Temozolomide (TMZ) is a standard chemotherapeutic for GBM, but TMZ treatment benefits are compromised by chemoresistance. This study aimed to elucidate the crucial mechanisms leading to EGFRvIII and TMZ resistance.

**Methods:**

CRISPR-Cas13a single-cell RNA-seq was performed to thoroughly mine EGFRvIII function in GBM. Western blot, real-time PCR, flow cytometry, and immunofluorescence were used to determine the chemoresistance role of E2F1 and RAD51-associated protein 1 (RAD51AP1).

**Results:**

Bioinformatic analysis identified E2F1 as the key transcription factor in EGFRvIII-positive living cells. Bulk RNA-seq analysis revealed that E2F1 is a crucial transcription factor under TMZ treatment. Western blot suggested enhanced expression of E2F1 in EGFRvIII-positive and TMZ-treated glioma cells. Knockdown of E2F1 increased sensitivity to TMZ. Venn diagram profiling showed that RAD51AP1 is positively correlated with E2F1, mediates TMZ resistance, and has a potential E2F1 binding site on the promoter. Knockdown of RAD51AP1 enhanced the sensitivity of TMZ; however, overexpression of RAD51AP1 was not sufficient to cause chemotherapy resistance in glioma cells. Furthermore, RAD51AP1 did not impact TMZ sensitivity in GBM cells with high O^6^-methylguanine-DNA methyltransferase (MGMT) expression. The level of RAD51AP1 expression correlated with the survival rate in MGMT-methylated, but not MGMT-unmethylated TMZ-treated GBM patients.

**Conclusions:**

Our results suggest that E2F1 is a key transcription factor in EGFRvIII-positive glioma cells and quickly responds to TMZ treatment. RAD51AP1 was shown to be upregulated by E2F1 for DNA double strand break repair. Targeting RAD51AP1 could facilitate achieving an ideal therapeutic effect in MGMT-methylated GBM cells.

## Introduction

Cancer has become the leading cause of death in China^1^. Gliomas are the most common primary brain tumor and are characterized by rapid malignant progression, poor response to radiotherapy and chemotherapy, high recurrence and mortality rates, and a poor prognosis^2–6^. Currently, the first-line orphan chemotherapy drug for glioma clinical treatment is temozolomide (TMZ), but some patients are not responsive to TMZ and responsive patients may develop drug resistance after treatment, thus limiting the clinical efficacy of TMZ^7,8^.

TMZ is a DNA alkylating agent. Two metabolism steps are required for TMZ to exert an anti-tumor role after entering the cell^9^. TMZ is hydrolyzed and metabolized to 3-methyl-(triazen-1-yl)imidazole-4-carboximide (MTIC), then metabolized to 5-aminoimidazole-4-carboxamide (AIC) and diazomethane. Diazomethane is an effective component that can cause DNA guanine O^6^ (O^6^-MeG) site methylation^10^. O^6^-MeG can then cause DNA strand breakage and replication fork collapse, leading to cell death^11,12^. The DNA repair enzyme, O^6^-methylguanine-DNA methyltransferase (MGMT), can repair O^6^-MeG to normal guanine, which eliminates the chemotherapeutic effect of TMZ^13^. MGMT expression is regulated in gliomas by methylation of the promoter region. Therefore, it is generally recognized that the methylation level of the MGMT promoter region predicts the prognosis of patients after TMZ chemotherapy^14–17^; however, some patients with hypermethylation of the MGMT promoter region are not responsive to TMZ, and some TMZ-resistant cell lines have low MGMT expression, suggesting that other TMZ resistance mechanisms exist^18^.

A deficiency in mismatch repair (MMR) and enhanced DNA damage repair (DDR) systems have been observed in MGMT^low^ TMZ-resistant glioma cells^19,20^. Without effective repair, TMZ-induced DNA lesions eventually lead to DNA strand breaks (DSBs)^21^. Homologous recombination (HR) and non-homologous end-joining (NHEJ) are two major mechanisms involved in DSB repair. HR repair has a vital role in glioma TMZ resistance, which is consistent with the G2/M cell cycle arrest caused by TMZ^18,22^.

The transcription factor, E2F1, has important functions in cell proliferation, chromosome instability, and the DNA damage response^23^. Currently, the E2F family consists of eight members. E2F1, E2F2, and E2F3a are transcriptional activators, while E2F3b and E2F4∼8 are transcriptional repressors^24^. These factors share a similar DNA binding sequence (TTCCCGCC) and form a protein complex with TFDP1-3 to perform functions as a dimer^23^. E2F1 also promotes DNA repair factor recruitment to DSB sites and the acetylation of H3K9, suggesting a complex function of this protein^25,26^. E2F1 promotes tumor progression in hepatocellular carcinoma, breast cancer and renal cell carcinoma^27–29^. E2F1 has been reported to be upregulated by PDGF in gliomas and supports pro-neural glioma progression by enhancing USP1 expression^30^. Several reports consider E2F1 as a drug resistance factor for TMZ by default; however, a profile of E2F1 function under TMZ treatment is needed.

In this study we identified E2F1 as a rapidly upregulated transcription factor in response to TMZ treatment. RAD51-associated protein 1 (RAD51AP1) is a DDR-related protein that facilitates RAD51 to perform HR function^31^. We previously reported that RAD51AP1 is correlated with glioma clinical grades and has an oncogenic role in GBM^32^. We further demonstrated that RAD51AP1 is a mediator of E2F1 in TMZ resistance and that targeting RAD51AP1 has great potential in sensitizing glioma cells to TMZ.

## Materials and methods

### Cell culture, siRNA, and lentivirus transfection

U87-MG and T98G cells were cultured in DMEM (catalog # PM150210; Procell, Wuhan, China) containing 10% fetal bovine serum (Hakata, Shanghai, China) and 1% penicillin–streptomycin (Gibco, San Diego, CA, USA). TBD0220^33^ and N33^34^ primary glioma cell lines were cultured in complete DMEM/F12 (catalog # PM150312; Procell). The cells were incubated at 37°C in 5% CO_2_.

The siRNAs for DP1 were ordered from GenePharma (Suzhou, China) and transfected into glioma cells by Lipofectamine 3000 (Thermo Fisher Scientific, Waltham, MA, USA) according to the manufacturer’s instructions.

Lentiviruses containing EGFRvIII, E2F1, and si-E2F1 were purchased from CENECHEM (Shanghai, China). Lenti-Tet-On-RAD51ap1 and Lenti-Tet-On-shRAD51AP1 were obtained from Integrated Biotech Solutions (Ibsbio, Shanghai, China). The Tet-On system was induced by doxycycline (MCE, South Brunswick Township, NJ, USA). The sequence of siRNAs was provided in **Table 1**.

**Table 1:** siRNAs and primers

NC siRNA: UUCUCCGAACGUGUCACGUTT
siDP1-1: CUACGGCAUUUCUCCAUGATT
siDP1-2: GACGAUGACUUCAACGAGATT
si-E2F1-1: GCTGGACCACCTGATGAAT
si-E2F1-2: GGACTCTTCGGAGAACTTT
shRAD51AP1: GCAGTGTAGCCAGTGATTA
E2F1-F: GGATTTCACACCTTTTCCTGGAT
E2F1-R: CCTGGAAACTGACCATCAGTACCT
RAD51AP1-F: ATGACAAGCTCTACCAGAGAGAC
RAD51AP1-R: CACATTAGTGGTGACTGTTGGAA
GAPDH-F: TGCACCACCAACTGCTTAGC
GAPDH-R: GGCATGGACTGTGGTCATGAG
CHIP-RAD51AP1-F1: TACGGAAAAGCGGTGCCTT
CHIP-RAD51AP1-R1: ATTCTTGTTGGCTTCAGCGG
CHIP-RAD51AP1-F2: GGAGGGCGGGAGCAAATTC
CHIP-RAD51AP1-R2: AATTCTTGTTGGCTTCAGCGG

### Western blot

Cells were dispensed in 6-well plates. After treatment, cells were washed with ice-cold phosphate-buffered saline (PBS) and lysed in RIPA buffer containing protease inhibitors (Solarbio, Beijing, China) for total protein extraction. The cell lysates were incubated on ice for 30 min, and shocked for 30 s every 10 min. A nuclear and cytoplasmic protein extraction kit (Beyotime, Beijing, China) was used to extract nuclear and cytoplasmic protein fractions. After measuring protein concentrations with a bicinchoninic acid kit (Solarbio), equal amounts of protein were subjected to SDS-PAGE, then transferred to polyvinylidene fluoride membranes and incubated with primary antibodies overnight at 4°C. The blots were incubated with horseradish peroxidase-conjugated secondary antibody and detected using a ProteinSimple FluorChem M system (ProteinSimple, Silicon Valley, CA, USA). The primary antibodies included anti-E2F1 (catalog # 3742S; CST, Boston, MA, USA), anti-H3 (catalog # 17168-1-AP; Proteintech Group, Wuhan, China), anti-GAPDH (catalog # 60004-1-Ig; Proteintech Group), anti-DP1 (catalog # ab186831; Abcam, Cambridge, UK), anti-γ-H2A.x (catalog # ab2893; Abcam), and anti-MGMT (#86039; CST).

### Real-time PCR

Total RNA was extracted using TRIzol reagent (Vazyme, Nanjing, China). A GoScript™ reverse transcription reagent kit (Promega, Madison, WI, USA) was used to synthesize cDNAs according to the manufacturer’s instructions. Real-time PCR performed in triplicate by ABI QuantStudio3 (Thermo Fisher Scientific). Gene expression was normalized to GAPDH and the relative value of gene expression was calculated as 2^-ΔΔct35^. The primers were provided in **Table 1**.

### Immunohistochemistry (IHC) staining

IHC staining was performed on intracranial tumors by subjecting 5-μm paraffin sections to a three-step process using a DAB staining kit (Zsgb-Bio, Beijing, China), as previously reported^33^.

### Cell viability assay

Cell viability assays were performed using Cell Counting Kit-8 (CCK8; MCE). Briefly, 2 × 10^3^ cells were cultured in 96-well plates with at least 5 multiple wells for every sample. TMZ (Selleck, TX, USA) was added 24 h after cells were plated. At the end of the experiment, 10 μL of the CCK-8 solution was added to each well and the converted dye absorbance was measured at 450 nm with a microplate reader (Synergy 2; BioTek Instruments, Winooski, VT, USA).

### Flow cytometry (FACS)

Cells were dispensed in 6-well plates and treated with TMZ for 3 days. The cells were then collected and fixed with 70% ethanol for 24 h at 4°C, and the cell cycle was analyzed by Beijing Dingguo Changsheng Biotechnology Co. Ltd. (Beijing, China). The cells were collected and resuspended in 400 μL of ice-cold PBS for apoptosis analysis. An apoptosis detection kit (BestBio, Shanghai, China) was used to stain the cells, and Annexin-V FITC and 7-AAD were detected using a BD Calibur flow cytometer (BD Biosciences, Franklin Lakes, NJ, USA).

### Immunofluorescence (IF) staining

Cells were cultured in confocal dishes and treated with TMZ 24 h after plating. The cells were fixed with 4% paraformaldehyde [PFA] (Solarbio) for 20 min at room temperature (between 20°C and 27°C), and permeabilized with 0.1% Triton X-100. The cells were blocked in 5% BSA (Solarbio) for 1 h at room temperature. The γ-H2A.x primary antibody (catalog # 9718S; CST) was incubated overnight and detected with Alexa Fluor 594-conjugated secondary antibody (catalog # A-21207; Life Technologies, Grand Island, NY, USA). Images were obtained using a FluoView 1200 system (Olympus, Tokyo, Japan). The same confocal scanning parameters were maintained across samples and the images were minimally processed to maintain data integrity.

### Chromatin immunoprecipitation (ChIP) assay

The ChIP assay was performed using the Millipore Magna ChIP™ A/G Chromatin Immunoprecipitation kit (catalog # 17-10085; billerica, MA, USA) according to the manufacturer’s instructions as previously reported^36^. The precipitated DNA fragments were quantified by real-time PCR analysis.

### Establishment of the intracranial glioma model

Female BALB/c nude mice (4–6 weeks old) were purchased from the Animal Center of the Cancer Institute of the Chinese Academy of Medical Science (Beijing, China). TBD0220 GBM cells (5 × 10^5^) were stereotactically-injected into the right corpus striatum of the nude mice. TMZ (5 mg/kg) was administered by gavage as follows: administration for 5 d; and weekly withdrawal for 2 d for 4 weeks. The mouse experiments were performed according to the protocols approved by the Animal Ethical and Welfare Committee of Tianjin Medical University General Hospital [approval no. ZYY-DWFL-IRB-002(F)-01].

### Single cell and statistical analysis

Single cell RNA-seq was performed as described in our previous work^37^. The dual-ended sequencing mode of the 10X single cell application and Illumina sequencing platform were used to perform high-throughput sequencing on samples. The 10X internal software (CellRanger) was used to perform data quality statistics on the original data and compare the reference genome annotated with the Ensembl reference gene. The software quantified the high-throughput single cell transcriptome by identifying the barcode sequence markers that distinguish cells in the sequence and the UMI markers that identify different mRNA molecules in each cell. The quality control method used in the initial analysis process was as follows: cells with reserved cell gene and UMI numbers within the range of ± 3 times of the average standard deviation, and the mitochondrial gene ratio < 30% were regarded as high-quality cells for downstream analysis. Because the number of cells captured by each sample was < 10,000 and in the subsequent process of cell type identification no unclear identification was found, the same cell expressed multiple cell type markers.

Statistical analyses were performed using GraphPad (GraphPad Software, San Diego, CA, USA) or R software version 3.5.3 (The R Foundation, Vienna, Austria). Unpaired Student *t*-tests were used for comparisons between two groups. Statistical significance was set at a *P* < 0.05.

## Results

### E2F1 is a key transcription factor for living cells and chemostress

**Figure 1:**
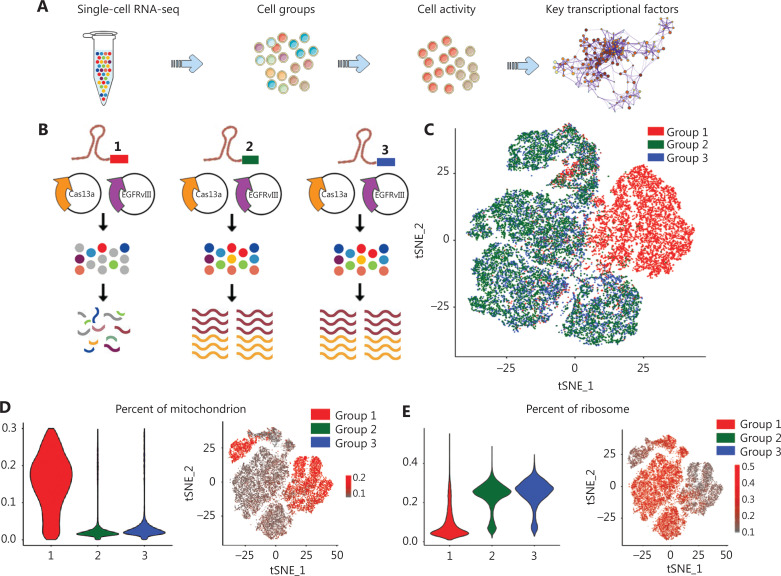

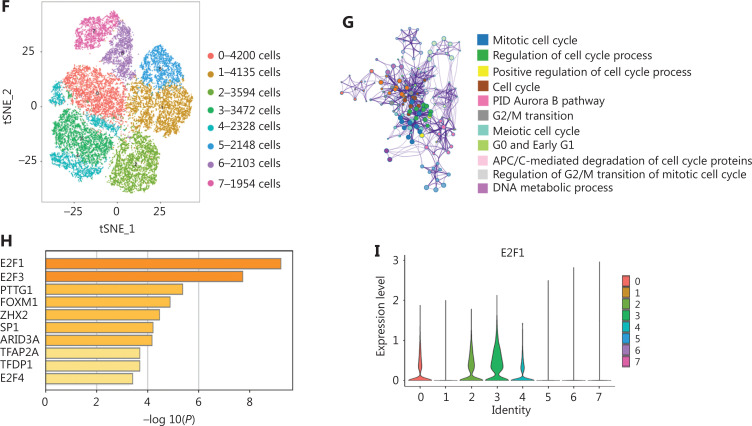
E2F1 is among the most important transcription factors in EGFRvIII cells. (A) Scheme of single-cell RNA-seq and transcription factor analysis. (B) Schematic representation of the CRISPR–Cas13a induced-collateral effect in EGFRvIII-positive glioma cells. (C) tSNE analysis of CRISPR–Cas13a treated cells. (D) The percentage of mitochondrial mRNAs was profiled by generating violin and tSNE plots. (E) The percentage of ribosome mRNAs was profiled by generating violin and tSNE plots. (F) The cluster of each cell is visualized as a tSNE plot. (G) GO analysis of representative genes in EGFRvIII living cells. (H) TTrust analysis was used to profile key transcription factors in EGFRvIII living cells. (I) Distribution of E2F1 in single-cell clusters is shown as violin plots.

We previously reported that the CRISPR–Cas13a system induces collateral effects in U87 glioma cells^37^. To further analyze the characteristics of these cells, we used CellRanger 3.0 to quantify the high-throughput single-cell transcriptome by identifying the barcode sequence and UMI markers of different mRNAs in each cell. The original data were annotated based on the Ensembl reference genome. Then, the cells were grouped by treatment and cell activity status, and the key transcription factors of differential genes were analyzed. The summary of the process is presented in **Figure 1A**. Cells with collateral effects were referred to as Group 1 (red), invalid EGFRvIII crRNA treatment was classified as Group 2 (green), and Lipofectamine 3000 only was classified as Group 3 (blue; **Figure 1B**). tSNE cluster analysis revealed that cells in Group 2 were highly coincident with cells in Group 3. The Group 1 cells exhibited a completely different distribution state (**Figure 1C**). Mitochondria play a fundamental role in the regulation of eukaryotic cell life. Mitochondrial-mediated apoptosis increases mitochondrial permeability^38^. Thus, we assumed that the content of mitochondrial mRNA in Cas13a-triggered apoptotic cells is higher than unedited cells. The violin plot analysis revealed that > 15% of the transcripts in Group 1 was mitochondria mRNA, and 2%∼3% in Groups 2 and 3. The feature plot analysis also revealed high expression of mitochondrial genes in Group 1 (**Figure 1D**). The percentage of ribosome genes was much lower in Group 1 cells, which is consistent with the degradation of ribosomes we previously reported^37^ (**Figure 1E**). Then, the cells were placed in eight clusters based on tSNE visualization. The Group 1 cells were mainly in Clusters 1 and 5. The cells in Groups 2 and 3 were distributed in Clusters 0, 2, 3, 4, 6, and 7 (**Figure 1F**). The cells in Clusters 0, 2, 3, and 4 were unedited and active, as shown in **Figure 1D and 1E** (low mitochondria and high ribosome transcripts). CD24 (Cluster 0), PDPN (Cluster 2), CCNE1/E2F8 (Cluster 3), and CDC25C/DLGAP5/HMMR (Cluster 4) were in the top 10 marker genes of the clusters, which coincided with the good condition of these cells (**Supplementary Figure S1**). Gene Ontology (GO) analysis revealed pathway enrichment in the cell cycle and mitosis (**Figure 1G**). TTrust analysis^39^ demonstrated that E2F1 and E2F3 were the most significant TFs for upregulated genes, which further confirmed the activity of the cell cycle-related pathway in active cells (**Figure 1H**). We analyzed the expression of E2F1 and showed that E2F1 was expressed at higher levels in living EGFRvIII cells than in cells with collateral effects, indicating an important role of E2F1 in maintaining cell activity (**Figure 1F**). Then, we re-analyzed the RNA-seq data of U87vIII cells treated with TMZ for 14 d^40^ (**Supplementary Figure S2A**). The top 1,000 genes with the most significant upregulation were enriched in processes related to DNA replication, the E2F pathway, and apoptotic signaling pathways (**Supplementary Figure S2B**). E2F1 was the most active transcription factors under TMZ treatment (**Supplementary Figure S2C**).

**Figure 2:**
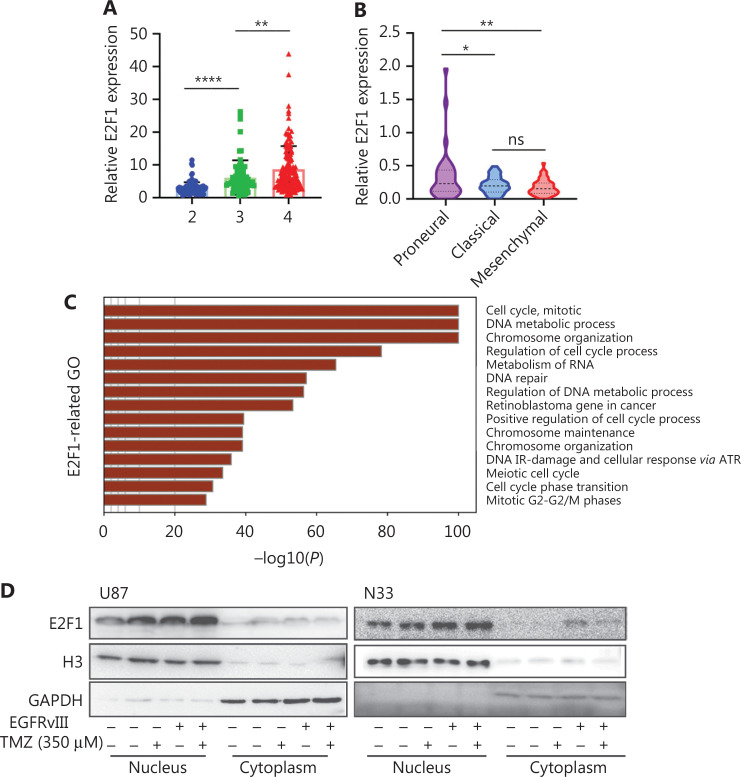

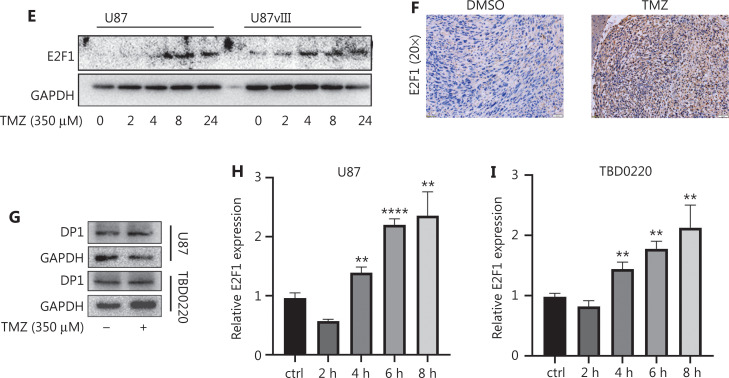
E2F1 is a TMZ-response transcription factor. (A) The expression of E2F1 in different WHO grades was calculated and visualized as scatter plots. (B) The expression of E2F1 in different GBM subtypes was visualized as violin plots. (C) Major functions of E2F1-positive related genes were profiled by GO analysis. (D) Western blot analysis was used to examine the expression of E2F1 under EGFRvIII and/or TMZ treatment. (E) E2F1 expression under TMZ time course treatment was tested by Western blot. (F) IHC staining was used to estimate E2F1 expression in DMSO- or TMZ-treated mouse glioma tissues (bar = 50 μM). (G) DP1 expression after TMZ treatment was examined by Western blot. Real-time PCR analysis was used to determine the time course of E2F1 mRNA expression under TMZ treatment in U87 (H) and TBD0220 (I) cells. *P < 0.05, **P < 0.01, and ****P < 0.0001.

We profiled E2F1 expression in the CGGA database^41^ and found that E2F1 was upregulated as the clinical grade increased (**Figure 2A**), and obtained the highest expression in the pro-neural GBM subtype (**Figure 2B**). GO analysis indicated that the cell cycle, mitosis, and DNA repair response were enriched in E2F1-positive related genes (**Figure 2C**). To further verify the role of E2F1, we examined E2F1 protein expression after EGFRvIII overexpression and/or TMZ treatment. Western blot analysis revealed that E2F1 mainly existed in the nucleus and was upregulated by EGFRvIII expression and TMZ treatment (**Figure 2D**). The level of E2F1 protein was upregulated by TMZ within 4 h, demonstrating a rapid response to TMZ (**Figure 2E**). We analyzed E2F1 expression in an intracranial glioma model by IHC staining and showed that TMZ significantly increased E2F1 protein levels *in vivo* (**Figure 2F**). DP1 is a partner of the E2F family, cooperating with E2Fs to perform functions^42^. We examined DP1 expression after TMZ treatment and showed that DP1 expression was not impacted by TMZ (**Figure 2G**). We analyzed the expression of E2F1 mRNA in glioma cells To study the regulation of EGFRvIII and TMZ on E2F1 to study EGFRvIII and TMZ regulation of E2F1. Real-time PCR analysis revealed that EGFRvIII and TMZ upregulated E2F1 mRNA levels, suggesting a transcriptional regulation mechanism (**Figure 2H, 2I**). Furthermore, TMZ enhanced E2F1 mRNA expression within 4 h, which is consistent with E2F1 as an element of fast response to TMZ damage.

### E2F1-mediated TMZ resistance *via* RAD51AP1

**Figure 3:**
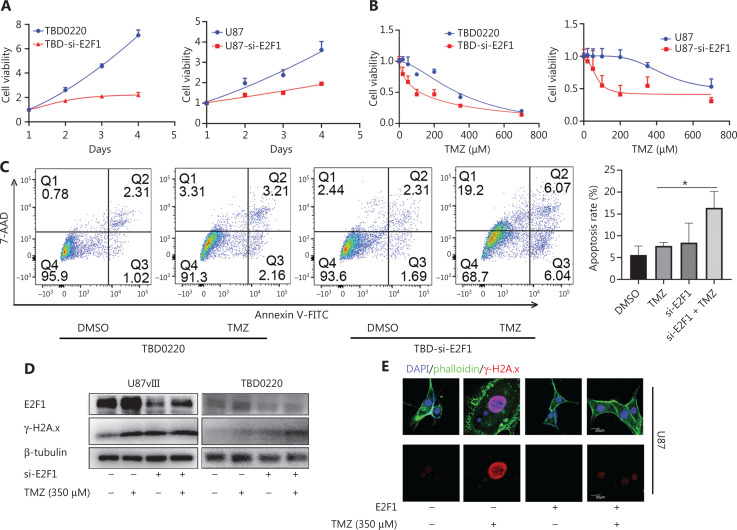
E2F1 promotes TMZ resistance in glioma cells. (A) Cell proliferation rates of TBD0220 and U87 cells with or without E2F1 knockdown were determined by CCK8 analysis. (B) TBD0220 and U87 cells with or without E2F1 knockdown were treated with the indicated dose of TMZ for 3 d, and sensitivity to TMZ was determined by CCK8 analysis. (C) Apoptosis of TBD0220 cells with or without E2F1 knockdown was determined by FACS analysis. (D) Western blot analysis was used to evaluate E2F1 and γ-H2A.x expression under the indicated treatment. (E) U87 cells with or without E2F1 overexpression were treated with TMZ for 1 d, and γ-H2A.x foci were measured by IF analysis. The value of the bar is the comparison between TMZ and siE2F1+TMZ groups. *P < 0.05.

We knocked down E2F1 by shRNA to analyze the role of E2F1 in cell proliferation and TMZ resistance, and showed that cell viability was significantly reduced (**Figure 3A**). Knocking down E2F1 also enhanced sensitivity to TMZ (**Figure 3B**). We then analyzed the role of E2F1 in TMZ-induced glioma cell apoptosis. FACS analysis indicated that knocking down E2F1 had a nominal impact on TBD0220 cell apoptosis, but significantly increased apoptosis of cells under TMZ treatment (**Figure 3C**). Western blot analysis indicated an enhanced DNA DSB based on upregulation of γ-H2A.x in shE2F1 cells under TMZ treatment (**Figure 3D**). Then, we transfected U87 cells with lenti-E2F1. Confocal analysis confirmed less severe DNA damage induced by TMZ after E2F1 overexpression (**Figure 3E**).

**Figure 4:**
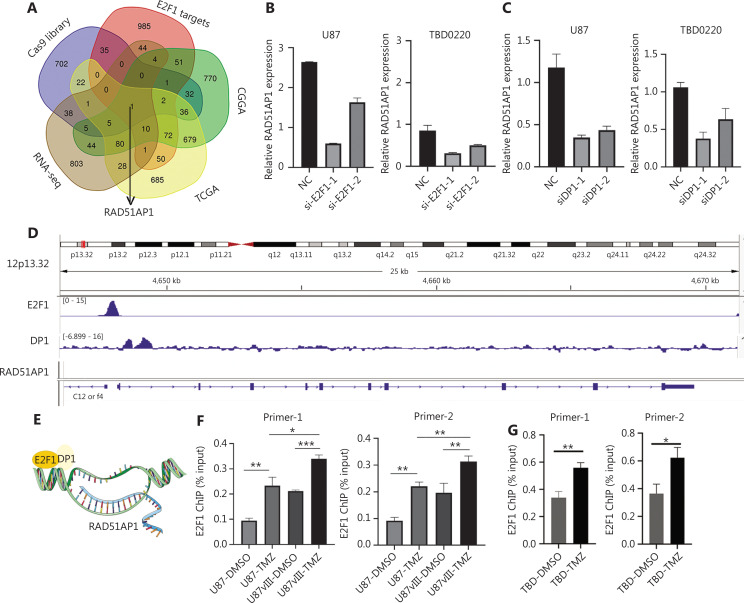

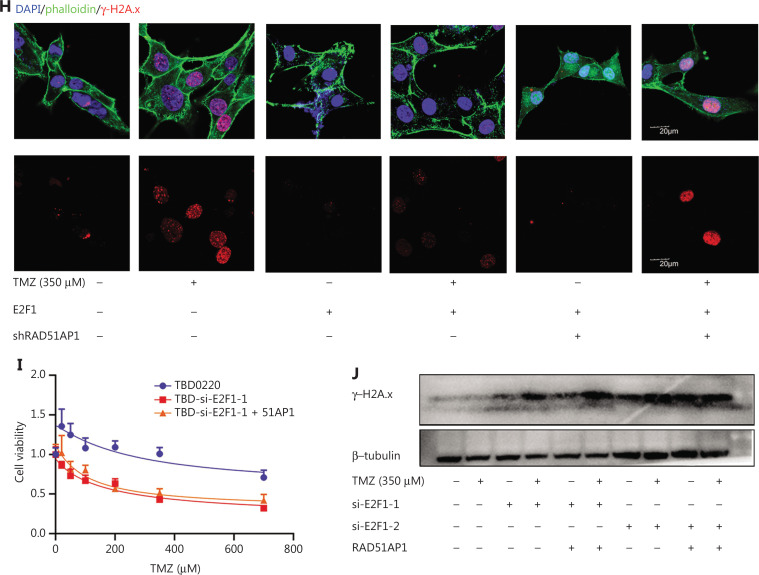
RAD51AP1 is an effector of E2F1 for TMZ resistance. (A) The Venn diagram shows differentially-expressed genes for the indicated groups; RAD51AP1 is the only gene among the five groups. Real-time PCR analysis was used to examine the expression of RAD51AP1 after knocking down E2F1 (B) or DP1 (C) in glioma cells. (D) The E2F1 binding peak on the genome location of RAD51AP1 was profiled by Integrative Genomic Viewer (IGV). (E) The scheme by which E2F1 upregulates RAD51AP1. ChIP-PCR analysis was used to evaluate E2F1 binding to the promoter of RAD51AP1 in U87 (F) and TBD0220 (G) cells. (H) U87 cells were transfected with E2F1 overexpression and/or shRAD51AP1 lentivirus, and γ-H2A.x foci after TMZ treatment were measured by IF analysis (bar = 20 μM). (I) TBD0220 cells were transfected with si-E2F1-1 and/or Tet-On RAD51AP1 lentivirus, and sensitivity to TMZ was determined by CCK8 analysis. (J) TBD0220 cells were transfected with si-E2F1s and/or Tet-On RAD51AP1 lentivirus, and γ-H2A.x expression under TMZ treatment was measured by Western blot.

E2F1 is a key transcription factor that regulates various genes. We analyzed E2F1-positive related genes in the CGGA and TCGA databases, E2F1 target genes, TMZ treated RNA-seq data, and lethal genes in the CRISPR–Cas9 library to investigate important genes that mediated E2F1-induced TMZ resistance. Venn diagram analysis indicated that RAD51AP1 was the only gene based on different filter conditions (**Figure 4A**). Knocking down E2F1 and DP1 significantly reduced the level of RAD51AP1 mRNA (**Figure 4B, 4C**). We analyzed the ChIP-seq data of E2F1 in U87 cells^43^ and identified an enrichment site (chr12:4,647,800-4,648,200) in the promoter of RAD51AP1 (**Figure 4D**). We employed ChIP PCR analysis to further confirm the transcriptional activation of RAD51AP1 by E2F1. Both TMZ and EGFRvIII enhanced the binding of E2F1 to the promoter region of RAD51AP1, indicating a direct enhancement in U87 and TBD0220 cells (**Figure 4F, 4G**). We constructed U87-E2F1^OE^-Tet-On-shRAD51AP1 cells to examine if targeting RAD51AP1 could reverse the TMZ resistance effect of E2F1. Immunofluorescence analysis revealed that E2F1 overexpression reduced the γ-H2A.x foci after TMZ treatment, while knockdown of RAD51AP1 reversed this effect (**Figure 4H**); however, overexpression of RAD51AP1 did not effectively increase the cell viability caused by E2F1 knockdown under TMZ treatment (**Figure 4I**). Furthermore, Western blot analysis confirmed failure of RAD51AP1 to reverse the E2F1 knockdown phenotype (**Figure 4J**).

**Figure 5:**
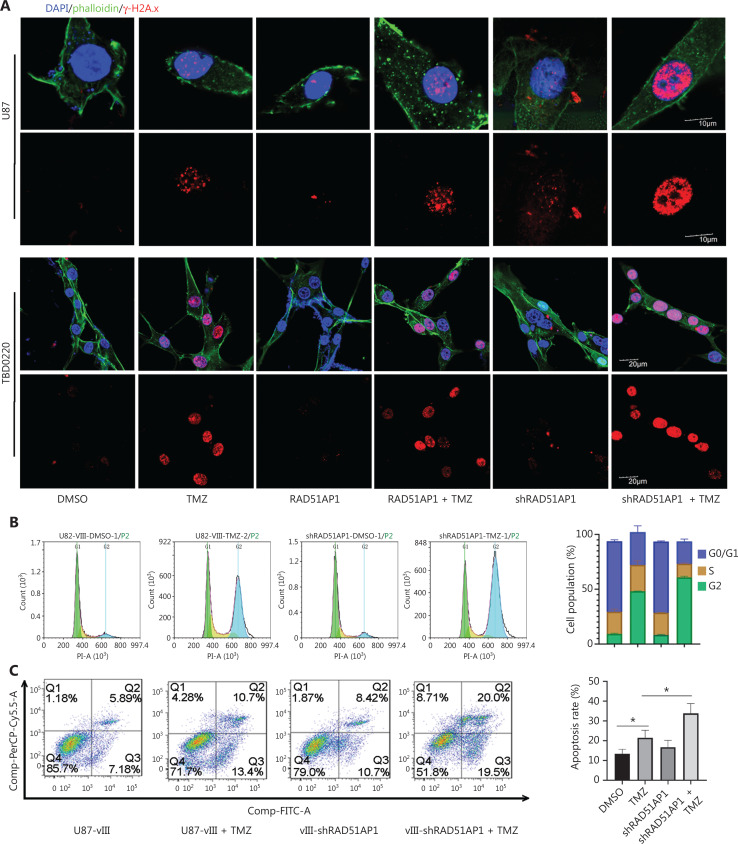

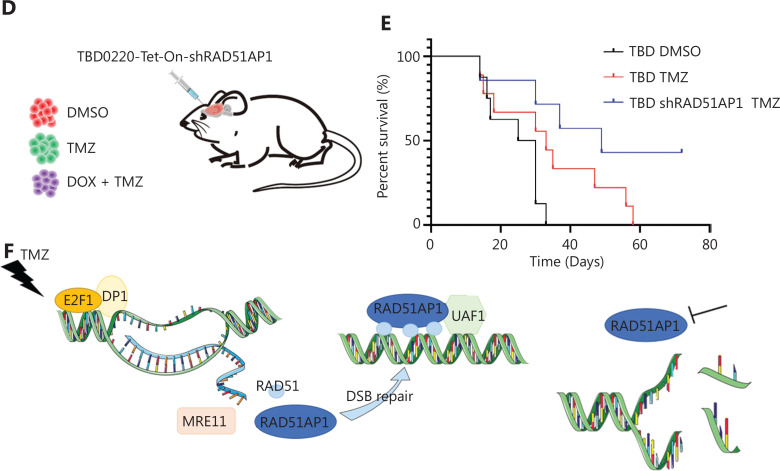
Targeting RAD51AP1 enhanced TMZ sensitivity in glioma cells. (A) U87 and TBD0220 cells were transfected with RAD51AP1 overexpression or shRAD51AP1 lentivirus, and γ-H2A.x foci after TMZ treatment were measured by IF analysis. Cell cycle arrest (B) and apoptosis (C) of U87vIII cells under the indicated treatment were analyzed by FACS. (D) Scheme of the grouping of glioma tumor models. Dox (10 mg/kg) was dissolved in water. TMZ (5 mg/kg) was given to mice by gavage. The mode was 5 days of administration and 2 days of withdrawal. The administration time was 4 weeks. (E) The survival curve for mice. (F) The mechanism underlying E2F1-RAD51AP1 axis involvement in TMZ resistance of glioma cells.

We then performed immunofluorescence analysis to evaluate the role of RAD51AP1 in TMZ-induced DNA damage. TMZ treatment caused DNA DSBs in glioma cells, as indicated by increased γ-H2A.x foci. Overexpression of RAD51AP1 had a minimal impact on the chemoresistance of glioma cells; however, knockdown of this gene significantly increased the degree of DNA damage (**Figure 5A**). Cell cycle analysis revealed that knocking down RAD51AP1 enhanced TMZ arrest at the G2 stage, indicating an increased failure of HR repair (**Figure 5B**). FACS analysis further confirmed enhanced apoptosis under low levels of RAD51AP1 (**Figure 5C**). We previously reported that knocking down RAD51AP1 significantly improves survival in a mouse glioma model *in vivo*^32^. We constructed an intracranial glioma model with TBD0220 cells to further test and verify the role of RAD51AP1 in TMZ resistance *in vivo* (**Figure 5D**). Compared to the TMZ-treated group, knocking down RAD51AP1 further prolonged mouse survival, thus targeting RAD51AP1 could be a promising means for improving chemotherapy efficacy (**Figure 5E**). Because HR repair is carried out by complex protein, including RAD51, RAD51AP1, and UAF1^44^, we concluded that RAD51AP1 is necessary for TMZ-induced DSB repair; however, overexpression of RAD51AP1 alone is not sufficient to complete the repair work (**Figure 5F**).

### RAD51AP1 is essential for MGMT-methylated glioma TMZ resistance

**Figure 6:**
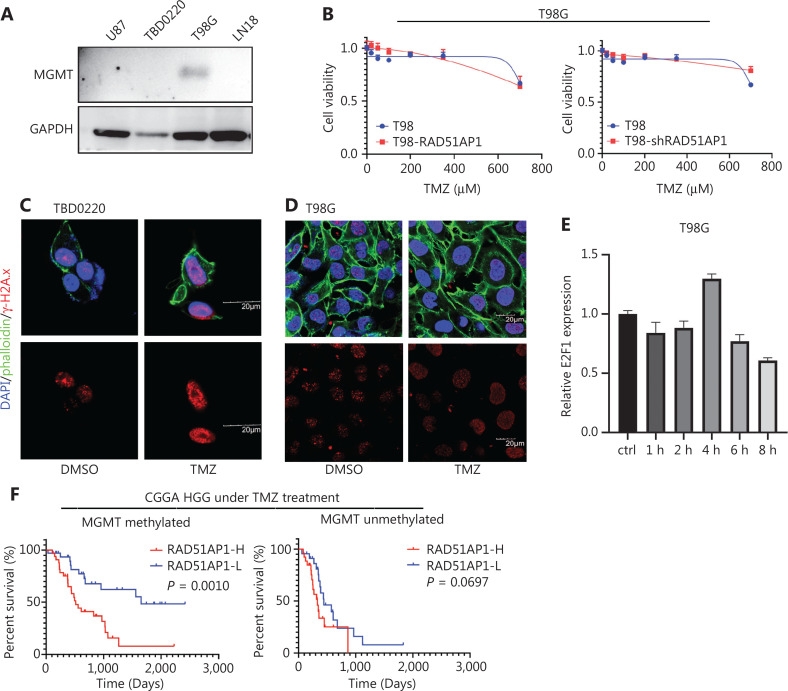
RAD51AP1 is a therapeutic target of TMZ resistance in MGMT-methylated gliomas. (A) MGMT expression in different glioma cells was determined by Western blot. (B) TMZ sensitivity of T98G cells with RAD51AP1 overexpression or knockdown was measured by CCK8 analysis. TBD0220 (C) and T98G (D) cells were treated with TMZ for 1 d, and γ-H2A.x foci were measured by IF analysis (bar = 20 μM). (E) Real-time PCR analysis was used to determine the time course of E2F1 mRNA expression under TMZ treatment. (F) Survival analysis of patients with different levels of RAD51AP1 expression and MGMT methylation in the CGGA database.

**Figure 7:**
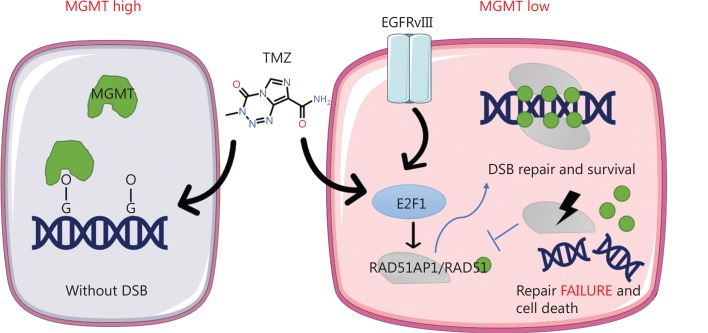
The scheme by which EGFRvIII and TMZ upregulates E2F1 to mediate chemoresistance. TMZ is a first-line chemotherapy drug for glioma. In MGMT-high gliomas, O^6^-MeG is repaired by MGMT without DNA double strand breaks. In MGMT-low and/or EGFRvIII-positive gliomas, E2F1 is upregulated by EGFRvIII and TMZ, then induces RAD51AP1 expression. RAD51AP1 facilitates RAD51 to repair the DNA double strand breaks caused by TMZ. Targeting RAD51AP1 could be an effective TMZ sensitization method.

MGMT is a critical chemoresistance factor that promotes O^6^-methylguanine DNA damage repair induced by TMZ^45^. Therefore, we determined if RAD51AP1 impacted the sensitivity of TMZ in cells with high levels of MGMT expression. Western blot analysis indicated that U87 and TBD0220 cells had low MGMT expression, while T98G is a GBM cell line with a high level of MGMT expression (**Figure 6A**). CCK8 analysis revealed that neither overexpression nor knockdown of RAD51AP1 had an impact on TMZ sensitivity in T98G cells (**Figure 6B**). Immunofluorescence analysis suggested that the increase in γ-H2A.x foci within T98G cells was much less under TMZ treatment compared to TBD0220 cells (**Figure 6C, 6D**). This finding coincided with the MGMT-mediated TMZ resistance mechanism. Furthermore, TMZ treatment did not induce rapid upregulation of E2F1 mRNA, implying a low level of DNA damage in T98G cells (**Figure 6E**). We analyzed the survival rate of glioma patients in the CGGA database and found that RAD51AP1 is a better prognostic factor in MGMT-methylated patients (**Figure 6F**). Overall, these data indicated that RAD51AP1 facilitated DSB repair in MGMT-methylated glioma cells and could be a therapeutic target in this type of glioma (**Figure 7**).

## Discussion

In this study we identified E2F1 as a key transcription factor in living EGFRvIII cells. Additionally, E2F1 was shown to be a rapidly upregulated transcription factor for TMZ resistance and enhanced DDR-related gene expression, including RAD51AP1 (**Figure 6G**).

We previously reported that CRISPR–Cas13a triggers collateral effects in glioma cells, leading to RNA degradation and cell death^37^. Re-analysis of the single-cell RNA-seq further confirmed upregulation of mitochondrial RNA and downregulation of ribosomal RNA in Cas13a-induced collateral U87vIII cells. Compared to the collateral effect cells, the living cells in Clusters 0, 2, 3, and 4 were enriched in the cell cycle and mitosis, and E2F1 was identified as a key transcription factor (**Figure 1**). In fact, E2F1 expression was higher in EGFRvIII-positive cells (**Figure 2D**).

Based on an analysis of U87vIII TMZ-treated bulk RNA-seq data we found that DNA replication, the E2F pathway, and ATR-related pathways were enriched in upregulated genes, supporting the important role of DDR in TMZ resistance among MGMT^low^ glioma cells^19^. Furthermore, E2F1 is a core transcriptional regulator of TMZ-induced genes, suggesting a key role of E2F1 in cell proliferation and chemoresistance (**Supplementary Figure S1**). The RNA-seq samples were treated with TMZ for 14 d. In addition, we found that E2F1 is upregulated in TMZ treatment within 4 h, indicating that E2F1 is a quick response and long-lasting transcription factor for chemoresistance. It has been reported that CHK2 activates E2F1 in response to DNA damage within 2 h in a posttranscriptional manner^46^. In the current study we showed that TMZ enhanced the levels of E2F1 mRNA and protein at 4 h, suggesting both transcriptional and posttranscriptional effects of E2F1 from this chemotherapy.

As a well-known transcription factor, E2F1 regulates various DDR-related genes^47^. We previously reported that RAD51AP1 is a potential mediator of EGFRvIII and an oncogene in GBM^32^. In the present study we further demonstrated that E2F1 directly binds to the promoter of RAD51AP1, and that this binding was enhanced in the presence of EGFRvIII and/or TMZ. Thus, the EGFRvIII-E2F1-RAD51AP1 axis might partially explain the TMZ resistance characteristics of EGFRvIII glioma cells^40^. As the ChIP-seq data (GSM4288488) indicated, DP1 was also located near, but not completely overlapping with E2F1 in the RAD51AP1 promoter. Because E2F1 was regulated by TMZ and DP1 was minimally impacted by this treatment, we concluded that E2F1 has a key role in RAD51AP1 regulation, as least in TMZ-resistant glioma cells. We showed that overexpression of RAD51AP1 alone was not sufficient to mediate TMZ resistance, which is reasonable because DNA damage repair requires coordination of multiple recombinants; however, RAD51AP1 could be an effective therapeutic target due to its important role in the DNA damage repair complex (**Figures 4, 5**). In addition, knockdown of E2F1 affects various genes because it is a key transcription factor, while targeting RAD51AP1 may have less unwanted effect on cells. Furthermore, RAD51AP1 is a better predictor of TMZ in MGMT-methylated clinical patients, implying an important role of HR repair in TMZ resistance in this category of patients (**Figure 6**).

## Conclusions

We demonstrated that E2F1 is a key transcription factor in EGFRvIII-positive glioma cells and profiled the functions of E2F1 in TMZ resistance. RAD51AP1 is a necessary mediator of E2F1 for glioma chemoresistance. Targeting RAD51AP1 could therefore facilitate achieving an ideal therapeutic effect in MGMT methylated-GBM cells.
