# Supplementary material for: Identification of the E2F1-RAD51AP1 axis as a key factor in MGMT-methylated GBM TMZ resistance
Source: Cancer Biol Med. 2023 Jun 5;20(5):385–400. doi: 10.20892/j.issn.2095-3941.2023.0011 (PMC10246439; doi:10.20892/j.issn.2095-3941.2023.0011)
Supplement: Supplementary file 1 [file cbm-20-385-s001.pdf]

Supplementary materials

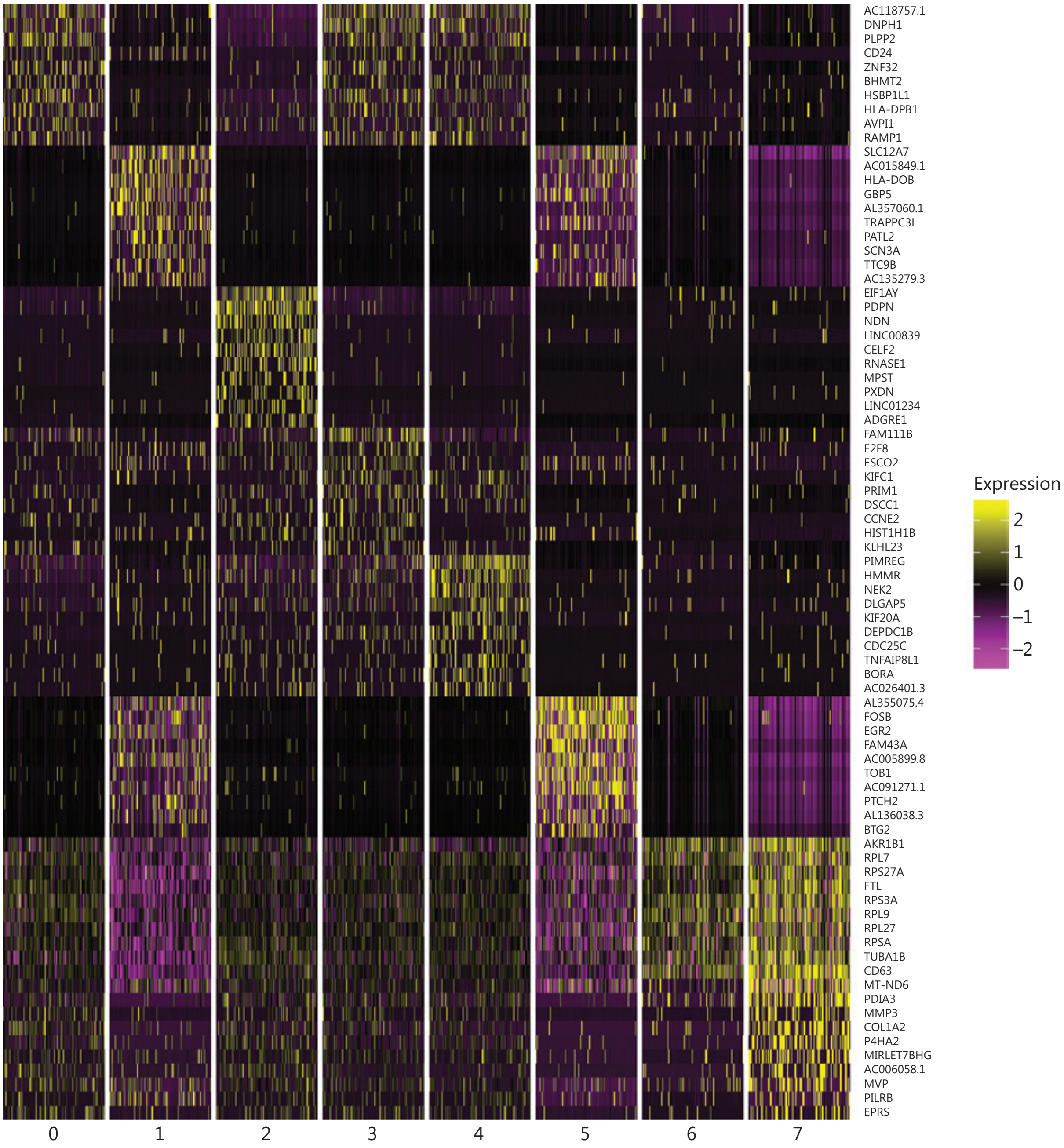

Figure S1 Expression profiles of the top 10 marker genes of each cluster are displayed as a heatmap.

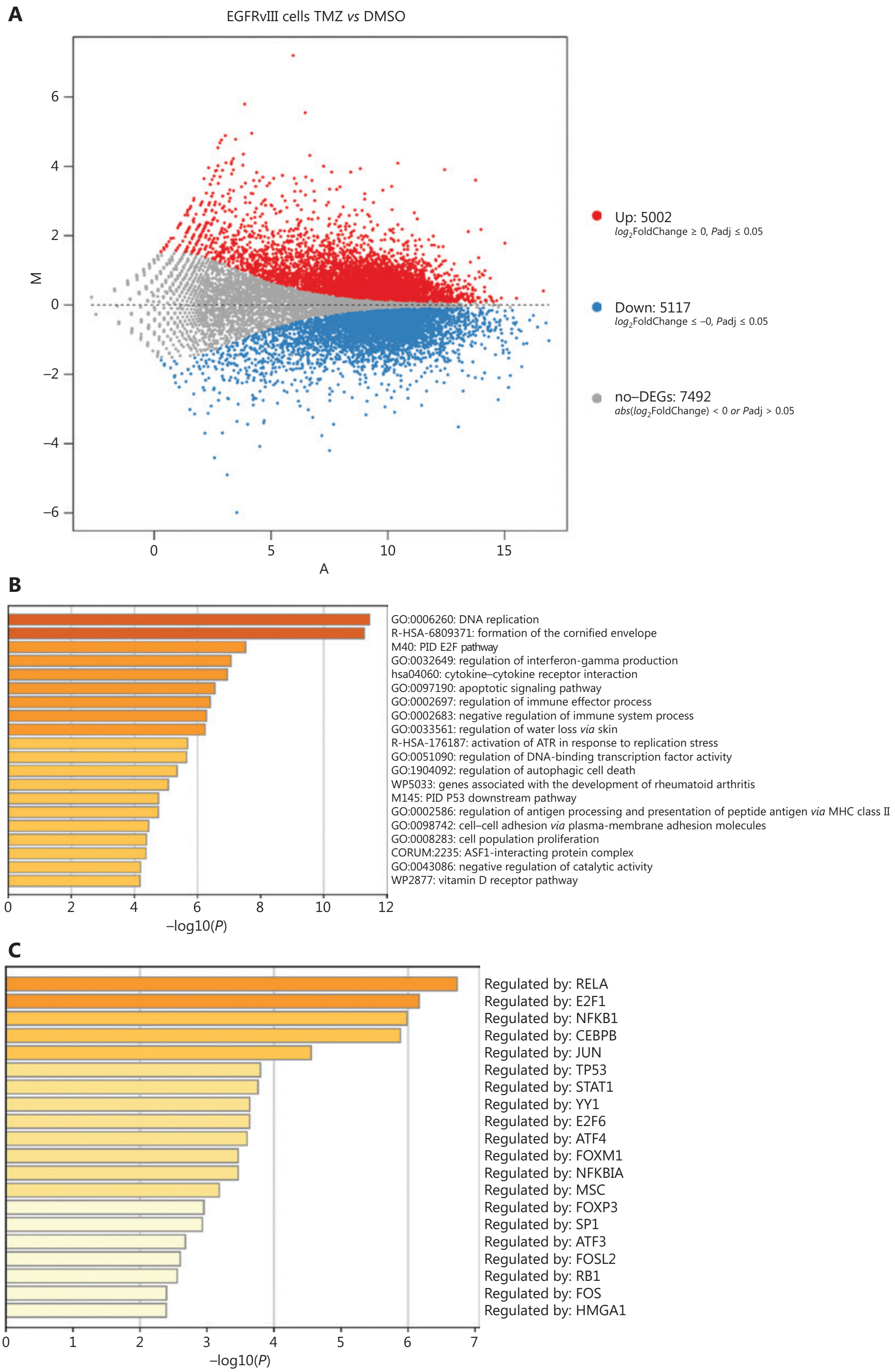

Figure S2 Profile of U87vIII cells treated with TMZ. U87vIII cells were treated with 350 μM TMZ for 14 d, and RNA-seq analysis was used to analyze gene expression. (A) The differentially-expressed genes are presented in volcano plots. (B) The enriched pathways after TMZ treatment. (C) Key transcription factors after TMZ treatment.
